# Fat Reduction in Peruvian Carrot (*Arracacia xanthorrhiza*) Snacks: Effectiveness of Edible Coatings and Optimization of Frying Conditions

**DOI:** 10.3390/foods14111895

**Published:** 2025-05-26

**Authors:** Viviane de Souza Silva, Luna Valentina Angulo Arias, José Ignacio Velasco, Farayde Matta Fakhouri, Rafael Augustus de Oliveira

**Affiliations:** 1 Poly2 Group, Department of Materials Science and Engineering, Universitat Politècnica de Catalunya (UPC BarcelonaTech), ESEIAAT, 08034 Terrassa, Spain; viviane.de.souza@upc.edu (V.d.S.S.); jose.ignacio.velasco@upc.edu (J.I.V.); 2 School of Agricultural Engineering, University of Campinas, Campinas 13083-875, SP, Brazil; lunavale@unicamp.br (L.V.A.A.); augustus@g.unicamp.br (R.A.d.O.)

**Keywords:** filmogenic solution, edible coating, fat reduction

## Abstract

Peruvian carrot is a root with a pleasant taste but a short shelf life. Developing Peruvian carrot snacks with appealing sensory characteristics, a crunchy texture, and reduced lipid content aligns with modern consumer demands and represents an innovative approach to food diversification. To ensure product quality, snacks must have a low water content to prevent microbial growth and maintain crispness. Therefore, optimizing process variables through pre-treatments is essential to achieving the desired characteristics. This study aimed to evaluate the influence of frying conditions on the water content, lipid absorption, and sensory acceptance of Peruvian carrot snacks. The preparation process involved sanitizing and slicing the roots, air-drying them at room temperature, and frying them according to an experimental design. The optimal frying conditions were 160 °C for 18 s and 174 °C for 30 s. Three different treatments were tested: two with edible coatings made from Peruvian carrot starch at concentrations of 3:20 and 5:10 (starch %/glycerol %) and one control sample without a coating. Sensory evaluation showed significant differences between coated and uncoated snacks, with all samples receiving high consumer acceptance. Notably, coated snacks exhibited a 50% reduction in lipid absorption compared to the control while also retaining a lower water content, key factors in maintaining texture, preserving quality, and extending shelf life. Furthermore, the application of edible coatings proved effective in reducing the caloric content of the snacks, making them a healthier alternative.

## 1. Introduction

Peruvian carrot (*Arracacia xanthorrhiza* Bancroft), or arracacha or mandioquinha, is an edible root from South America [1]. In Brazil, it is commercialized in many regions, especially in the south and southwest [2]. The Peruvian carrot root is rich in easily digestible carbohydrates [1], vitamin A, vitamin C, vitamin B3 [3], calcium, phosphorus [4], iron [5], and fiber [3]. Usually, it is commercialized in natura, but it is a perishable product with a short shelf life [1]. Therefore, the production of fast-food-like snacks is an alternative to incentivize Peruvian carrot production and turn it into a more accessible product for the market.

For the production of these snacks, the root is cut into thin slices and fried in vegetable oil. The snacks’ shape and size are directly related to oil absorption after the frying process, as thinner snacks have greater water loss, resulting in a crunchy texture, which is a desired characteristic for this type of food.

In the frying process, the raw material of a product is immersed in hot oil (with temperatures ranging between 160 °C and 190 °C [6]), resulting in a dried cooked product. The frying process also provides the particular organoleptic characteristics of color, taste, and texture that promote wide acceptance by consumers [7]. The frying temperature is a parameter linked to the quality aspect of the oil and the final product. According to Brazilian Resolution RDC nº 216 of 15 September 2004 [8], the temperature must not exceed 180 °C [9,10,11] in order to guarantee the quality and stability of the vegetable oils. Indeed, if temperatures above 200 °C are used, they promote isomerization reactions and the formation of trans fatty acids [11].

Throughout the frying process, soybean oil is less absorbed by potato chips compared to sunflower and corn oils [12]. According to Cella, Regitano-D’Arc, and Spoto [13], during the frying process of vegetables (potatoes, cauliflower, zucchini, and cassava), soybean oil showed thermal stability between 170 and 180 °C. According to Jorge et al. [14], in the frying process of potato chips, the durability of soybean oil after 6.5 h of heating was similar to that of sunflower oil and greater than that of corn oil. In the work conducted by Dodoo et al. [15] involving the production of yam fries at 175 °C, soybean oil underwent less oxidation than sunflower oil but more than coconut oil.

The composition of oils is directly linked to health-related concerns, such as cardiovascular diseases and obesity. According to Márquez-Ruiz, Velasco, and Holgado [16], to ensure greater oxidative stability during frying, oils should contain less than 3% linolenic acid (ω-3), more than 40% oleic acid, and less than 50% linoleic acid (ω-6) due to their susceptibility to oxidation reactions.

As reported by Abrante-Pascual, Nieva-Echevarría, and Goicoechea-Oses [11], soybean oil contains between 4.5 and 11.0 mg/kg of linolenic acid (ω-3), 17.0 to 30.0 mg/kg of oleic acid, and 48.0 to 59.0 mg/kg of linoleic acid (ω-6), making it more prone to chemical degradation due to the nature of its fatty acid bonds. These contents are linked to chemical degradation due to the nature of its fatty acid bonds. Therefore, the adoption of good frying practices, such as working with temperatures below 180 °C, pre-drying treatments, and minimizing oil reuse, can significantly reduce the formation of undesirable and harmful compounds [11].

The demand for ready-to-eat food products, mainly fried ones, has increased along with the growth of the working population [17] due to lacking time to prepare a meal. However, these ready-to-eat food products, especially fried ones, are not always considered healthy alternatives. This is why a reduction in the fat content of these foods is one of the greater challenges of the food industry [7,18], focusing on the prevention of the incidence of health problems, such as obesity and overweight [19], which are related to the consumption of this type of food. Thus, Peruvian carrot snacks must be sensorily attractive, with a crunchy texture and a low fat content. Therefore, optimization methods and pre-treatments are applied to improve the conditions of the frying process.

A central composite rotatable design (CCRD) is an experimental design that allows users to work with a fewer number of combinations between the levels of the factors studied, given a surface response capable of predicting the interaction of the variables and showing the optimal point or region for a specific condition [20]. This includes temperature and time conditions under which the process parameter could reduce the fat content. In addition, the application of edible coatings to snacks before the frying process could help to reduce fat absorption, and consequently the caloric content of the product [9,21,22,23,24]. This type of coating could be produced from natural polymers (proteins, polysaccharides, and fat), where the prepared solutions are applied directly to the food, forming a thin layer. This thin layer, after the drying process, can provide protection to the coated product, increase its shelf life, and preserve its quality [19].

Finally, the aim of this study was to verify the influence of temperature and time in the frying process, which is related to the water content, fat absorption, and sensory acceptance (color) of Peruvian carrot snacks. In addition, this study states the optimized frying condition prior to the coating application.

## 2. Materials and Methods

### 2.1. Raw Material

The Peruvian carrot root was obtained from the CEASA (Centrais de Abastecimento de Campinas S.A., Campinas, São Paulo, Brazil). The material was previously chilled in a cold chamber at 7 °C and processed the same day it was delivered.

### 2.2. Peruvian Carrot Root Preparation (Snack Preparation)

#### 2.2.1. Peruvian Carrot Root Sanitization

The root was washed with water using soft bristle brushes to remove soil residues. Then, the root was peeled, immersed in sodium hypochlorite (Ypê, Amparo, São Paulo, Brazil) with a dilution of 15 mL/L for 15 min, and washed again with distilled water. Finally, the root was sliced using a food processor (PA-7, Skymen, São Paulo, SP, Brazil), with the thickness fixed at around 3 mm.

#### 2.2.2. Frying the Peruvian Carrot Root 

The Peruvian carrot root was fried in an electric frier (Roto Fry Branco FB895, DeLonghi, Treviso, Italia) with temperature controls. The proportion used for frying was 1:8 (p/p); therefore, 150 g of Peruvian carrot root was fried in 1200 g of refined soy oil, in such a way that the slices were submersed evenly. After the frying process, the excess oil was removed by centrifugation at 2300 rpm for 3 min (Arno, São Paulo, SP, Brazil).

#### 2.2.3. Frying Process Optimization

For the experimental design, time and temperature were the independent variables, according to Rodrigues and Iemma [20], using a central composite rotatable design (CCRD). A total of 11 experiments were run, with 4 axial and 3 central points, to develop predictive mathematical models for water content and fat absorption.

For temperature, the maximum level was established according to Brazilian legislation (the Technical Regulation of Good Practices for Food Service, resolution 216 of 15 September 2004 [8]), which determines a maximum of 180 °C for the use of oils and fat. The temperature ranged from 140 to 180 °C, and the time ranged from 18 to 102 s.

### 2.3. Edible Coating Preparation and Application

#### 2.3.1. Film-Forming Solution Preparation

The procedure proposed by Fakhouri [25] for edible coating preparation was considered, with modifications, for the film-forming solution starch of Peruvian carrot, and the manual stirring time was 4 min and 30 s. The starch and glycerol (P.A., Synth, Diadema, SP, Brazil) were weighed in analytical balance (CE, AY220, Kyoto, Japan) to obtain a concentration of 3:20 (*w*/*w*) and 5:10 (*w*/*w*), measured in grams, respectively, for 100 mL of distilled water. The starch and the water were mixed in a 250 mL glass beaker until a homogeneous solution was obtained and brought to a thermostatic bath (Quimis, Q334 M-14, Diadema, SP, Brazil) at 70 °C. To improve coating homogenization, the film-forming solution was manually stirred for 4 min and 30 s, then removed from the thermostatic bath and mixed with glycerol.

#### 2.3.2. Coating Peruvian Carrot Root Slices 

After sanitizing and slicing the Peruvian carrot root, as described previously, the sliced root was immersed into the film-forming solution for 1 min. The control samples (without the coating) were immersed in distilled water for 1 min. Then, before the frying process, the root slices were placed separately in sieves at room temperature (25 °C) with an air conditioner for 36 h. For the frying process, the parameters for optimization were considered.

### 2.4. Quality Parameters Evaluated for the Peruvian Carrot Snacks

#### 2.4.1. Peruvian Carrot Snacks’ Water Content

The water content of Peruvian carrot snacks with and without a coating was determined according to method 926.12 of AOAC [26].

#### 2.4.2. Fat Content

The Instituto Adolfo Lutz method [27] for the determination of fat content was followed. The sample was continuously heated in an ether (Sigma-Aldrich, Vetec, Madrid, Spain) solution to extract the fat. After extraction, the ether was distilled off, and the fat remaining in the sample was dried in an oven at 105 °C for one hour. The fat content was determined by the difference in weight between the flask before and after extraction.

#### 2.4.3. Sensory Evaluation of Peruvian Carrot Snacks

The sensory evaluation was performed by untrained tasters and following the method described by Macfie et al. [28], with block balancing of all the samples from the experimental design (color acceptance) and the sensory attributes (global appearance, shininess, color, flavor, and texture). Samples were served monadically, coded with three random digits, and presented in white cups on a white table. In the evaluation, tasters ranked how much they liked or disliked the samples using a structured hedonic scale of nine points, with the extremes corresponding to: 1 (extremely disliked) and 9 (extremely liked). The 44 tasters signed a consent form to participate in the sensory analysis. The project was approved by the Ethics Committee (CAAE 46859215.2.0000.5404).

Prior to the coating treatments, to optimize the frying process, a visual evaluation of color was performed for the Peruvian carrot snacks. Then, to evaluate the sensory parameters of the coating treatments, besides color, global appearance, shine, flavor, and texture were evaluated.

#### 2.4.4. Color Assessment

The variation of the Peruvian carrot snacks after the frying process was measured with a colorimeter (Minolta, CR- 400 model, Tokyo, Japan). Color measurements were taken immediately after the frying process and subsequent cooling of the snacks. Color readings were obtained based on the CIELAB color system (L*, a*, and b*), and the ΔE parameter (total color difference) was used to quantify changes in the product color. Three independent measurements were taken for each experimental frying condition, ensuring the reproducibility and accuracy of the data obtained. For the blank, the sample before the frying process was used to determine the change in color after the process, in which the average results were L* 83.18, a* 2.29, and b* 33.46.

### 2.5. Statistical Analysis

For the optimization of frying conditions, a central composite rotatable design (CCRD) was used, with 11 runs, 4 axials, and 3 central points. For the evaluation of the frying conditions of coated snacks, we used a factorial design 2^2^ with 6 runs for 2 coating formulations vs. 2 frying conditions.

The software Statistica 9.0 (Statsoft, EUA) was used for data treatment. Analysis of variance (ANOVA) and the Tukey test were used to determine the optimization of the frying process and to compare the significance of the coating treatment effects on the Peruvian carrot snacks at a confidence level of 95%.

## 3. Results

### 3.1. Frying Optimization of Peruvian Carrot Snacks

It was possible to evaluate the most optimal temperature and time parameters for the frying process of Peruvian carrot roots. Using the responses of the central composite rotatable design (CCRD) for water content, fat, sensorial color, and Delta E (ΔE) (Table 1), we obtained the predictive mathematical models described in Equations (1)–(4). These models were calculated using the encoded values of x and y (Table 1).

Water content = 5.37 − 4.52x − 0.85x^2^ − 0.28y^2^ + 0.32xy (1)

Fat content = 9.16 − 2.84 × 2 − 0.50y − 1.4y^2^ (2)

Sensorial color = 5.6555 − 0.9297x − 1.0929y − 1.0875xy(3)

ΔE = 34.6609 + 5.4014y − 6.3495y^2^ (4)

A determination coefficient (R^2^) of 0.84 was obtained for the water content mathematical model, expressing that the model could be used to predict the water content behavior related to the temperature and time factors in the frying process. On the other hand, for the fat model, there was an R^2^ of 0.73, meaning that the model was also capable of predicting the fat absorption behavior. For the sensory acceptance of color, a coefficient of determination (R^2^) of 0.86 was obtained. In this model, only the linear factors were statistically significant when considering pure error. However, in Equation (3), the interaction of the factors was also included because it has significance at the SS residual. However, it was considered that the model can be predictive, but it is important to note that the experimental results depend on human perception and are subject to major variations and errors. On the other hand, the instrumental analysis of color, represented by the ΔE, showed an R^2^ of 0.86, resulting in a predicted model in which temperature variation has a significant influence on the color changes of the product.

According to Table 2, there were significant statistical differences for both variables: time and temperature.

**Figure 1:**
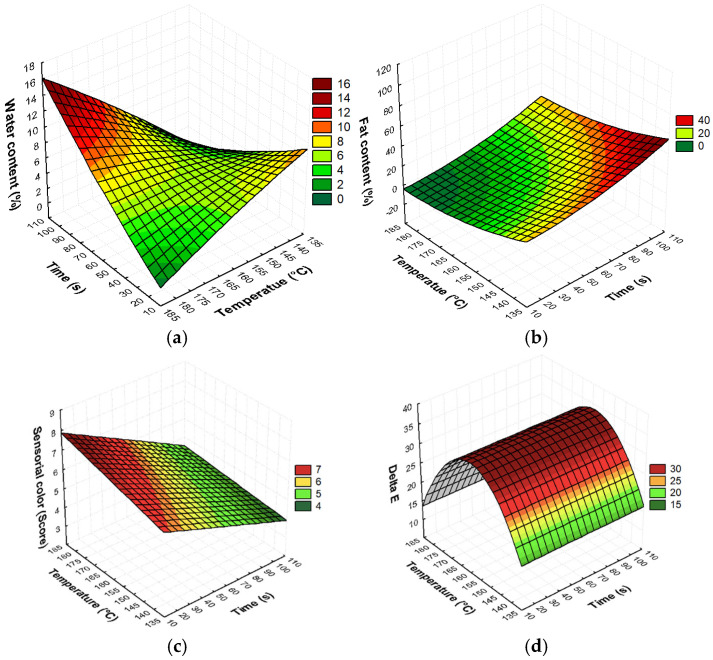
Response surfaces for (**a**) water content, (**b**) fat content, (**c**) sensorial color, and (**d**) Delta E.

The range of frying time was reduced by approximately 43% when compared with the work of Hua et al. [29], who used a frying process lasting 180 s. This is longer than 102 s, which was the higher frying time of the experimental design of this study. According to the results of the experimental design, it was possible to obtain regions of optimal conditions in the frying process using shorter times (Figure 1).

The raw material showed a water content of 78% (w.b.). Peruvian carrot snacks were dried at room temperature until the water content reached approximately 16% (w.b.) before the frying process. Because frying is also a drying process [30], there was also a reduction in the water content when compared to the initial value. However, the acceptance of the potential customer is an essential factor to consider.

According to the experimental design, when the snack is subjected to processing for a shorter time, it displays lower fat absorption, a reduced water content, and better color acceptability.

Thus, in order to achieve a lower water content with good sensorial acceptability, the best experimental treatment was number 3 (174 °C/30 s). For treatment 4 (174 °C/90 s), the results showed that it was possible to reduce the water content with a longer frying time, but the sensorial acceptability was affected, resulting in a low score (Table 1). This result can be predicted in the sensory analysis response surface (Figure 1c). Then, in treatment 6 (180 °C/60 s), the use of temperatures higher than 174 °C for 60 s of frying showed a similar water content to treatment 3, as well as a lower fat percentage; however, again, the sensorial acceptability was the lowest.

The highest experimental water content (10.17%) was shown in treatment 5 (140 °C/60 s), and the treatments with temperatures lower than 174 °C showed water contents higher than 4%. These results could be predicted according to the surface response (Figure 1a; Equation (1)). Therefore, the CCRD confirms that, by increasing the temperature during the frying process, it is possible to obtain snacks with a water content below 10% (Figure 1a).

It has been suggested that foods with a high water content can stimulate oil absorptions [31] as the water is probably replaced by the oil during the frying process [32,33]. However, according to the results (Table 1), different water contents (e.g., 2.11 and 10.17%) showed similar values in the quantification of fat (6.06 and 6.50%, respectively), demonstrating that other factors may be involved in the absorption of frying oil, such as the microstructure of the pores present in the snack and/or during cooling. As the food cools, the internal pressure of the pores increases during the frying process and decreases due to the condensation of water vapor. This favors a vacuum effect that draws the oil that is present on the surface into the pores [32]. However, according to Liu et al. [34], a drying pre-treatment can improve the texture of the fried root because it promotes dehydration and forms a crust that helps reduce oil absorption during the frying process when compared to the raw root. In addition, a low water content is related to food stability as it influences storage time, processing conditions, and packaging factors [35].

On the other hand, the fat content of the snacks ranged from 5.63 to 9.25% (Table 1), which is lower than the fried root snacks usually available at marketplaces, which have a fat content of about 30%. In order to confirm low oil absorption values, the nutritional information of commercial products available on the market was checked, showing that industrialized potato chips had a fat content of 35% [36] and baroa–potato chips (Peruvian carrot) had a fat content of 46% [37], referring to the portion (100 g) indicated on the packaging. Similar market values were also observed (Abdolshah [24]) for potato snacks, with fat absorption ranging from 25 to 42%. Therefore, if the target is to obtain products with lower fat and water contents, then the conditions of the frying process applied in this study allow us to optimize them.

Using Equation (2) and Figure 1b, it is possible to confirm the significant effect of both factors on fat content, but there was no significance for the interaction of temperature and time. The response surface (Figure 1b) shows that the highest fat absorption occurred at temperatures of 150 °C and below. For temperatures above 160 °C, fat absorption was reduced. However, Damy and Jorge [38], Jorge and Lunardi [12], and Kita et al. [39] also observed the effect of frying temperature on the reduction of water content and fat, but in potato snacks. They observed that the highest fat absorption values were present at lower temperatures. The same results were observed by Okon et al. [40] for cassava snacks.

### 3.2. Sensory Evaluation of Peruvian Carrot Snacks

Sensory tests evaluate the transformations that can interfere with the quality and acceptance of food products. They use the human senses as a measuring instrument and are important methods in the field of science and technology for studying the acceptability of various products [41]. There is equipment that is able to detect problems in food processing and storage. However, sometimes, they are unable to determine perceptible changes that affect consumer acceptance, as consumers want the food to have its characteristic color and texture. The sensory analysis carried out by evaluating visual appearance is fundamental, as it analyzes the first impressions of the product’s appearance, especially its color [42]. The sensory evaluation analyzed the Peruvian carrot snack’s acceptability by potential consumers, thus deducing the preference of a sample among the others analyzed and determining whether the product will be sold on the market.

According to Table 1, there were three statistically significant groups. More than 63% of the treatments had a sensorial color evaluation above 6, and the other samples had evaluation scores between 3 and 4.70. The worst color evaluations were related to higher frying times and temperatures (Table 1). Evaluation scores under 5 were considered unacceptable.

**Figure 2:**
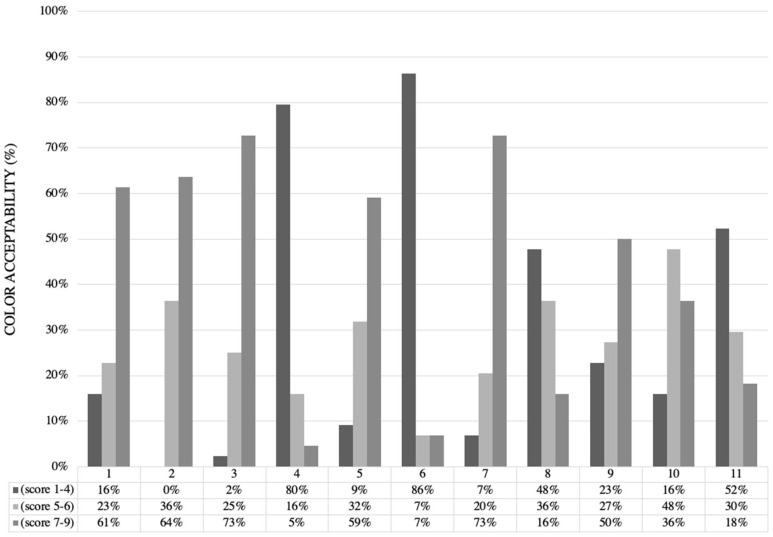
Color acceptability evaluation results for Peruvian carrot snacks after the frying process.

The color acceptability evaluation (Figure 2, Table 1) showed that out of 44 consumers, 72% liked it moderately (score 7) or liked it extremely (score 9) for treatments 3 (174 °C/30 s) and 7 (160 °C/18 s). Meanwhile, 9% of the consumers extremely and slightly disliked (scores between 1 and 4) the snacks produced using treatments 3 and 7. Therefore, 3 and 7 were the best treatments according to consumer experience. The most rejected snack samples were produced using treatment 6 (180 °C/60 s), which 86% of consumers extremely and slightly disliked.

According to Equation (3) and Figure 1c, the frying time and temperature show significant effects on the sensory acceptance of color. The negative coefficients associated with the main variables and their interaction indicate that simultaneously increasing the frying time and temperature results in a reduction in the sensory acceptance of the product’s color. The model suggests that ΔE (Equation (4), Figure 1d) shows two terms associated with the frying temperature, indicating that color variation is mainly linked to temperature variation.

By correlating both color results (sensorial and instrumental), it is possible to state that the predictive model of ΔE provides an objective and quantitative measure of changes in product color, while sensory analysis offers a subjective perspective of how these changes are perceived by the consumer. The ΔE model suggests that time has no significant effect on color, which is only dependent on temperature variation, while the sensory acceptance of color tends to decrease as time and temperature increase simultaneously.

In summary, both the sensory and predictive models are aligned in their results, with excessive color changes (detected by ΔE) negatively affecting sensory color acceptance. The correlation between these two methods highlights the importance of balancing the frying time and temperature to avoid visual changes that may be undesirable from both a technical and sensory point of view, guaranteeing the optimization of the quality of the final product.

Hence, considering the responses of the water content (which is related to crunchiness), low fat absorption, the sensory acceptance of color, and the overall color difference (ΔE), treatments 3 and 7 showed the best acceptability. Then, the frying conditions of treatments 3 and 7 were selected in order to obtain sensory acceptance of the snacks’ color that was higher than “slightly liked it” and a water content lower than 7%, considering the maximum limit of water content to obtain a crunchy product [43].

### 3.3. Frying Peruvian Carrot Snacks with an Edible Coating Made of Peruvian Carrot Starch

Considering the CCRD optimization and frying conditions for the evaluation of snack coatings, two formulations of the coating were applied and tested at two frying conditions, as shown in Table 3.

According to the water content results, all samples presented values under 7%, which is related to characteristics of crunchiness. In addition, there was a statistically significant difference among the samples, showing that the coated snacks subjected to frying conditions of 174 °C and 30 s had the lowest water content results. In addition, as previously observed, all snack samples presented lower fat absorption when compared to commercial products, but it was possible to obtain a lower value with snacks coated with 5% starch and 10% glycerol and fried at 160 °C for 18 s (treatment 5). Treatment 5 presented reductions of 31% and 50% for water and fat absorption, respectively.

Furthermore, all the coated samples showed water content and fat absorption reductions when compared to the control samples (Table 3). The reduction of fat absorption was also observed by Sothornvit [44] and reached 33% for banana snacks by using centrifugation and coating. Hua et al. [29] used a pectin coating to reduce fat absorption by 30% in potato chips. Yu et al. [45] coated potato chips with guar gum, showing a fat absorption reduction of 50%, and Gao et al. [46] achieved reductions of 27% to 43% in coated potato chips. Thus, within the literature and the results of this study, it is possible to confirm that one of the benefits of coatings is the significant promotion of fat absorption reductions in fried products.

### 3.4. Sensory Evaluation of Coated Peruvian Carrot Snacks

To evaluate the quality of the coated snacks, they were subjected to consumer experience testing by scoring the global acceptability, shininess, color, flavor, and texture (Table 4). In general, there was no statistically significant effect for time and temperature; therefore, those factors do not affect the snack’s quality as expected because of the optimization of the frying conditions prior to the coating treatments. However, the undercoated snacks in treatment 2 (174 °C/30 s) displayed a significant difference in global acceptability, shininess, and color, with the lowest scores. These results could be related to the darker color, as observed by Hua et al. [29], whose work showed that potato chips without a golden color presented the lowest scores. Hence, the results confirm the hypothesis that coatings help to improve the attributes of fried products.

**Figure 3:**
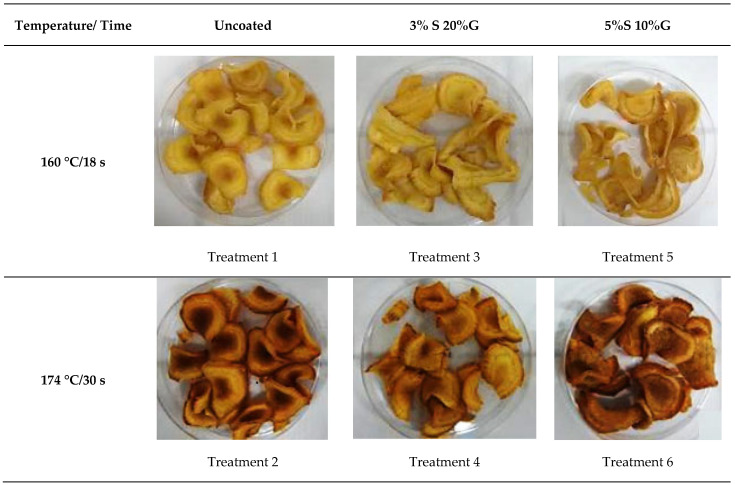
Photos of uncoated Peruvian carrot snacks and snacks with different coating formulations after frying under different process conditions. (S = starch; G = glycerol).

The samples with the best scores were the coated snacks with 5% of Peruvian carrot starch and 10% of glycerol, which were fried at 160 °C for 18 s (treatment 5). According to the sensory evaluations, treatment 5 achieved scores above 7 (liked it moderately) for global appearance, shininess, and color, and scores above 6 (slightly liked it) for flavor and texture. Then, when comparing the coated snacks with the control, there was no change in the acceptance of the sensory attributes, and the coating improved the consumer experience. This improvement could be related to a cross-link between the hydrocolloid and the plasticizer during gel formation, thus generating a resistant film on the surface of the snack [47], protecting it, and maintaining a more uniform visual color (Figure 3).

In general, considering the sensory attributes, the samples with the best results were the coated snacks subjected to frying conditions of 160 °C and 18 s (treatment 5), whose overall average scores were close to 7. Furthermore, the lower water content and lower lipid absorption results (Table 3) support the statement that the application of a starch-based Peruvian carrot coating helped to improve the sensory perception and nutritional quality of the Peruvian carrot snacks.

## 4. Conclusions

This study demonstrated the feasibility of producing Peruvian carrot snacks with desirable sensory characteristics, reduced fat content, and adequate moisture levels to ensure crispiness. Applying a central composite rotatable design (CCRD) enabled the optimal frying time and temperature conditions to be identified. The treatments with 174 °C/30 s and 160 °C/18 s were the most effective in achieving snacks with lower fat and moisture contents, as well as a high sensory acceptance of color.

Sensory analysis proved to be an essential tool for evaluating the acceptability of Peruvian carrot snacks, particularly in terms of color, an attribute that strongly influences consumer purchasing decisions. The results showed that the product’s visual appearance, which is heavily affected by frying conditions, directly impacts sensory acceptance. Comparing sensory data with instrumental color measurements (ΔE) revealed a significant correlation between excessive visual changes and product rejection by consumers, emphasizing that simultaneously increasing frying time and temperature negatively influences perceived quality.

Combining edible coatings with the optimized frying conditions determined by the central composite rotatable design (CCRD) proved to be an effective strategy for improving the quality of Peruvian carrot snacks. All coated samples exhibited water contents below 7%, indicating desirable crispiness. Notably, snacks coated with 5% starch and 10% glycerol and fried at 160 °C for 18 s achieved the most significant reductions in both water content (31%) and fat absorption (50%) compared to control samples, reinforcing their potential as a healthier alternative for consumers.

Therefore, careful selection of processing conditions, combined with complementary predictive and sensory tools, enables the optimization of Peruvian carrot snack quality, supporting its potential as an innovative and attractive product in the ready-to-eat food market.

However, the drying process for coated snacks remains a time-intensive step, highlighting the need for further research on alternative drying methods to enhance processing efficiency.

## Figures and Tables

**Table 1:** Central composite rotatable design (CCRD) results for Peruvian carrot snacks.

	Factors				Responses			
Treatment	Factor Level (x)	Time (s)	Factor Level (y)	Temperature (°C)	Water Content (%) *	Fat Content (%) *	Sensorial Color (Score)	Delta E (ΔE)
1	−1	30	−1	146	8.14 ± 0.10 ^b^	7.43 ± 0.10 ^d^	6.81 ^a^	26.05 ^bc^
2	1	90	−1	146	6.23 ± 0.09 ^d^	5.63 ± 0.08 ^g^	7.02 ^a^	21.51 ^cd^
3	−1	30	1	174	3.37 ± 0.10 ^h^	8.08 ± 0.09 ^c^	7.18 ^a^	37.74 ^ab^
4	1	90	1	174	2.11 ± 0.08 ^i^	6.06 ± 0.02 ^f^	3.04 ^c^	29.92 ^abc^
5	0	60	−1.414	140	10.17 ± 0.11 ^a^	6.50 ± 0.09 ^e^	6.63 ^a^	13.30 ^d^
6	0	60	1.414	180	3.66 ± 0.10 ^g^	6.62 ± 0.08 ^e^	3.00 ^c^	29.64 ^abc^
7	−1.414	18	0	160	4.84 ± 0.02 ^f^	7.35 ± 0.15 ^d^	7.09 ^a^	32.66 ^abc^
8	1.414	102	0	160	6.75 ± 0.06 ^c^	8.65 ± 0.03 ^b^	4.61 ^b^	31.24 ^abc^
9	0	60	0	160	5.40 ± 0.05 ^e^	9.21 ± 0.08 ^a^	6.09 ^a^	38.67 ^a^
10	0	60	0	160	5.30 ± 0.06 ^e^	9.03 ± 0.07 ^a^	6.04 ^a^	33.21 ^abc^
11	0	60	0	160	5.42 ± 0.09 ^e^	9.25 ± 0.09 ^a^	4.70 ^b^	36.53 ^ab^

(Means followed by the same letter have no significant difference). * Wet basis (w.b).

**Table 2:** ANOVA table for the results of Peruvian carrot snacks (water content and fat).

	SS	df	MS	F calc	F tab (5%)	*p*
Water content						
Regression	42.46	4	10.62	7.72	4.53	0.0005
Residue	8.25	6	1.38			
Lack of Fit	8.25	4	2.06	528.62	19.25	
Pure error	0.01	2	0.00			
Total	50.72	10				
**Fat**						
Regression	12.4	3	4.13	6.05	4.35	0.0001
Residue	4.78	7	0.68			
Lack of Fit	4.76	5	0.95	66.39	19.30	
Pure error	0.03	2	0.01			
Total	17.19	10				
**Sensorial color**						
Regression	21.20	3	7.07	13.9	3.07	0.08
Residue	3.56	7	0.51			
Lack of Fit	2.32	5	0.46	0.74	9.29	
Pure error	1.24	2	0.62			
Total	24.76	10				
**Delta E**						
Regression	482.63	2	241.31	23.86	4.46	0.03
Residue	80.90	8	10.11			
Lack of Fit	65.73	6	10.95	1.44	19.33	
Pure error	15.18	2	7.59			
Total	563.53	10				

SS: sum of square; df: degree of freedom; MS: mean of square; F: Fisher’s ratio; p: probability.

**Table 3:** Factorial design (2^2^) results for the water content and fat of coated Peruvian carrot snacks.

Treatment	Starch (%)	Glycerol (%)	Temperature (°C)	Time (s)	Water Content (%)	Water Content Reduction	Fat Content (%)	Fat Absorption Reduction
1	C	C	160	18	4.84 ± 0.02 ^a^		7.35 ± 0.15 ^a^	
2	C	C	174	30	3.37 ± 0.10 ^b^		8.08 ± 0.09 ^a^	
3	3	20	160	18	2.51 ± 0.11 ^c^	48%	5.52 ± 0.22 ^b^	25%
4	3	20	174	30	1.69 ± 0.16 ^d^	50%	5.17 ± 0.40 ^b^	36%
5	5	10	160	18	3.32 ± 0.39 ^b^	31%	3.65 ± 0.46 ^c^	50%
6	5	10	174	30	1.28 ± 0.19 ^d^	63%	5.50 ± 0.41 ^b^	32%

(Means followed by the same letter have no significant difference). C = control (not coated).

**Table 4:** Mean scores of the sensory attributes assigned by the evaluators to Peruvian carrot snacks (undercoated and coated).

Treatment	Global Appearance	Shininess	Color	Flavor	Texture
1	7.23 ^a^	7.15 ^a^	7.46 ^a^	6.15 ^a^	6.21 ^a^
2	5.06 ^b^	5.06 ^b^	4.92 ^b^	5.75 ^a^	6.38 ^a^
3	7.48 ^a^	7.27 ^a^	7.44 ^a^	6.40 ^a^	6.52 ^a^
4	6.08 ^a^	6.04 ^a^	6.27 ^a^	5.98 ^a^	6.54 ^a^
5	7.77 ^a^	7.54 ^a^	7.81 ^a^	6.60 ^a^	6.52 ^a^
6	6.56 ^a^	6.54 ^a^	6.48 ^a^	6.17 ^a^	6.44 ^a^

(Means followed by the same letter have no significant difference).

## Data Availability

The original contributions presented in the study are included in the article, further inquiries can be directed to the corresponding author.
